# A Comparative Analysis of Positive and Negative Stimuli for Takotsubo Cardiomyopathy: A Pooled Analysis of Two Studies and a Systematic Review

**DOI:** 10.7759/cureus.57816

**Published:** 2024-04-08

**Authors:** Arankesh Mahadevan, Vamsikalyan Borra, Lakshmi Prasanna Vaishnavi Kattamuri, Vikash Jaiswal, Ikechukwu R Ogbu

**Affiliations:** 1 Internal Medicine, SRM Medical College Hospital and Research Centre, Chennai, IND; 2 Internal Medicine, University of Texas Rio Grande Valley, Weslaco, USA; 3 Internal Medicine, Texas Tech University Health Sciences Center, El Paso, USA; 4 Research and Academic Affairs, Larkin Community Hospital, Miami, USA; 5 Cardiology, Sunrise Health GME Consortium, Las Vegas, USA

**Keywords:** pooled-analysis, risk-factors, takotsubo cardioyopathy, happy heart syndrome, broken-heart syndrome

## Abstract

Takotsubo cardiomyopathy (TTC) is characterized by transient myocardial dysfunction triggered by both negative and positive emotional experiences, known respectively as broken heart syndrome (BHS) and happy heart syndrome (HHS). Despite the scarcity of comparative analyses between HHS and BHS in the literature, our pooled analysis, incorporating two retrospective registry analyses of 1395 TTC patients (57 HHS and 1338 BHS), reveals that while BHS is more prevalent, both conditions exhibit similar clinical presentations and outcomes. Statistical analyses, utilizing binary random effects models, indicate that diabetes mellitus is less common in HHS patients and serves as a predictor for BHS. Furthermore, there are differences in cardiac imaging between the two groups; individuals with HHS have higher odds of experiencing midventricular ballooning, whereas those with BHS are more likely to have apical ballooning. These findings highlight the similarities in clinical features and outcomes between HHS and BHS, while also illustrating distinct imaging profiles. The study emphasizes the need for future prospective studies to delve deeper into the implications of these TTC subtypes, offering valuable insights into their comparative aspects and underlying mechanisms.

## Introduction and background

Stress cardiomyopathy is characterized by transient myocardial dysfunction, often mimicking acute coronary syndrome yet typically occurring without obstructive coronary artery disease [1]. This condition, generally known as Takotsubo cardiomyopathy (TTC), was first documented in a 1990 case report by Sato et al. [2]. The term is derived from the Japanese word for an octopus trap, alluding to the left ventricular ballooning observed during systole. TTC has been predominantly associated with acute emotional or physical stressors, with the latter being more prevalent. The stressors often have negative valence, such as bereavement, acute illness, or natural disasters, and have been commonly termed broken heart syndrome (BHS). However, one-third of patients may present without any preceding stressors [3].

Stress cardiomyopathy precipitated by a positive emotional experience, which is termed happy heart syndrome (HHS), was first documented in 2014 by Qin et al. [4], challenging the traditional association and thereby suggesting that both positive and negative triggers can precede stress cardiomyopathy. The progressively increasing prevalence of stress cardiomyopathy, coupled with the long-term mortality risk comparable to that of acute coronary syndrome, underscores the importance of accurate detection and management of this condition [1,5]. However, despite its clinical significance, a gap remains in comparative analyses of cardiomyopathic events triggered by positive versus negative emotional valences. Insights from two extensive registry studies have shed light on the potential differential impacts of this variation on patient presentation and outcomes [6,7].

Our pooled analysis aims to synthesize data from these studies, to enhance our understanding of TTC. Exploring the differences in cardiac complications and outcomes based on the nature of the preceding stimuli could potentially expand our current understanding of TTC, specifically the clinical differences between HHS and BHS.

## Review

Materials and methods

The study did not require ethical approval as it involved the analysis of published data; it adhered to the Preferred Reporting Items for Systemic Reviews and Meta-Analysis (PRISMA) guidelines.

Search and Screening Strategy

**Figure 1:**
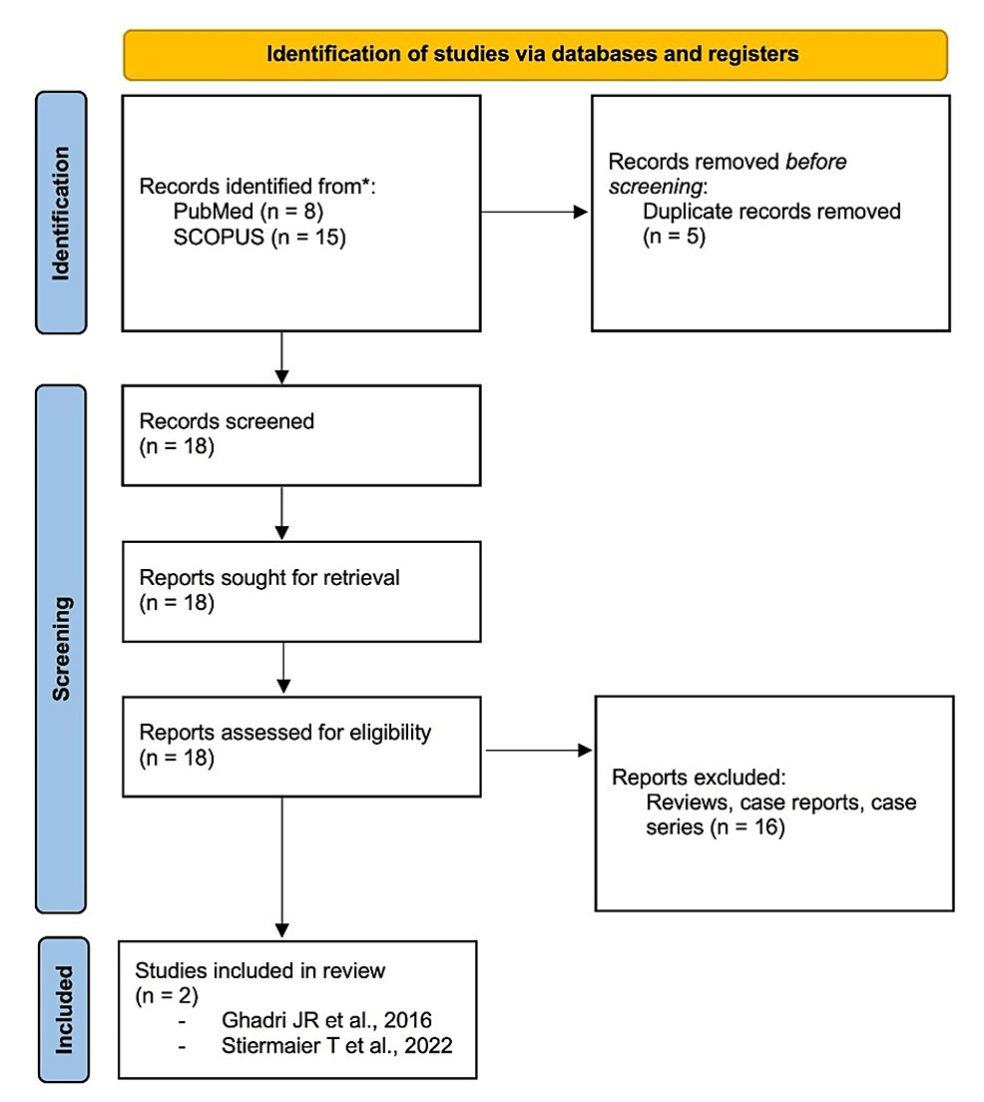
PRISMA chart depicting the search strategy* *[6,7]

We systematically searched PubMed and SCOPUS databases from their inception until October 2023 by using Medical Subject Headings (MeSH) and keywords in titles and abstracts. The search strategy included terms such as "Happy Heart Syndrome," "Takotsubo Cardiomyopathy," "Stress Cardiomyopathy," and "Broken Heart Syndrome," which were linked with Boolean operators. Titles and abstracts were independently screened for eligibility by two reviewers (VB and AM). We included studies presenting original comparative data on HHS and TTC, excluding meta-analyses, case reports, and reviews. Discrepancies were resolved through consensus between three reviewers. The PRISMA chart detailing the systematic search is presented in Figure 1.

Data Extraction and Analysis

Data extraction was independently conducted and verified by two reviewers (VB and AM). The extracted data encompassed study details (author, publication year, sample size, and design), participant demographics, clinical features, investigations conducted, and outcomes measured. The New-Castle Ottawa Scale (NOS) was employed for risk of bias assessment (Table 1). The quality of the included studies was assessed independently by two reviewers (VB and AM) and discrepancies were resolved by discussion. Statistical analyses in this meta-analysis were performed by using R software (Version 4.3.1, R Foundation for Statistical Computing, Vienna, Austria). A binary random-effects model was employed for calculating pooled unadjusted odds ratios (OR) with 95% confidence intervals (CI) and heterogeneity was assessed using the I² statistic. Statistical significance was established at a p-value of less than 0.05. Pooled analyses are displayed in forest plots.

**Table 1:** Newcastle-Ottowa Scale risk of bias assessment One asterisk (*) denotes 1 point; (-) denotes no points awarded. Score: 7–9: high quality; 4–6: high risk; and 0–3: very high risk of bias

Study	Selection				Comparability	Outcomes			Score
	Representativeness of the exposed cohort	Selection of the non-exposed cohort	Ascertainment of exposure	Demonstration that outcome of interest was not present at the start of the study	Comparability of cohorts based on the design or analysis	Assessment of outcome	Was follow-up long enough for outcomes to occur?	Adequacy of follow-up of cohorts	
Ghadri et al., 2016 [6]	*	*	*	*	-	*	*	*	7
Stiermaier et al., 2021 [7]	*	*	*	*	-	*	*	*	7

Results

Our systematic search identified 23 publications; after excluding five duplicates and 16 reports, which included reviews, case reports, and case series, two articles were selected for the final analysis (Figure 1).

The two primary studies included in the pooled analysis were those by Stiermaier et al. [7] and Ghadri et al. [6], encompassing 1395 patients with TTC. Of these, 57 were classified under HHS and 1338 under BHS. Of note, all included studies utilized the Mayo Clinic Diagnostic Criteria until 2016 and the European consensus criteria after 2016 for TTC. The differences regarding demographics, comorbidities, and clinical features between HHS and BHS groups are shown in Table 2.

**Table 2:** Overall baseline demographic and clinical characteristics ^a^Binary random effects model. ^b^Ghadri et al. [6] describe age (years) and ejection fraction using mean and SD. ^c^Stiermaier et al. [7] describe age (years) and ejection fraction using median and IQR. ^d^Based on the subset of the total population for whom data were available. ^#^Statistically significant at p<0.01 NR: not reported due to data unavailability; SD: standard deviation; IQR: Interquartile range

Characteristics		Overall (n=1395)	Happy heart syndrome (n=57)	Broken heart syndrome (n=1338)	Pooled analysis^a^, OR (95% CI)
Demographics					
Age (years)	Mean ± SD^b^	NR	71.4 ± 11.2	65 ± 12.5	-
	Median (IQR)^c^	NR	70 (61–77)	69 (62–78)	-
Sex	Male	77	8	69	-
	Female	1318	49	1269	0.37 (0.09–1.54)
Comorbidities^d^					
Hypertension		65.6%	56.1%	66%	0.61 (0.19–1.98)
Diabetes mellitus		14.3%	3.5%	14.8%	0.26 (0.07–0.94)
Hyperlipidaemia		39.9%	38.9%	40%	0.97 (0.55–1.72)
Smoking		16.9%	14%	17.1%	0.79 (0.37–1.70)
Clinical presentation ^d^					
Chest pain		81.8%	84.3%	81.7%	1.19 (0.55–2.58)
Breathlessness		33.5%	31.4%	33.5%	0.84 (0.27–2.57)
ST segment changes		70.2%	66.7%	70.4%	0.90 (0.23–3.48)
Ejection fraction (%)	Mean ± SD^b^	NR	40.2 ± 9.4	42.6 ± 11	-
	Median (IQR)^c^	NR	43 (31–45)	40 (35–45)	-
Cardiac ballooning pattern^d^					
Apical ballooning		84.2%	70.2%	84.8%	0.42 (0.23–0.75)^#^
Midventricular ballooning		13.1%	26.3%	12.5%	2.52 (1.36–4.68)^#^
Basal ballooning		1.8%	3.5%	1.7%	2.76 (0.72–10.58)
Outcome^d^: in-hospital mortality		1.2%	0	1.2%	-

Demographics and Comorbidities-Related Data

In the Stiermaier et al. study [7], the median age for BHS patients was 70 years (IQR: 61-77), while it was 69 years (IQR: 62-78) for HHS patients. In contrast, the Ghadri et al. study [6] reported the mean age of BHS patients as 65 years (SD: ± 12.5 years) and 71.4 years (SD: ± 11.2 years) for HHS patients. Regarding gender distribution, both HHS (86%) and BHS (95%) groups were predominantly female. However, the pooled analysis did not find female sex as a significant risk factor for HHS over BHS.

Diabetes mellitus was more prevalent in BHS (14.3%) than in HHS (3.5%) (OR: 0.26, 95% CI: 0.07-0.94), suggesting a lower risk of HHS among diabetics compared to BHS. The most common comorbidity was hypertension in both HHS and BHS (56% and 66%, respectively). Prevalence of hyperlipidemia and smoking in HHS and BHS were found to be 39% vs. 40% and 14% vs. 17%, respectively. Hypertension, hyperlipidemia, and smoking were not found to be significant risk factors for HHS.

Clinical Features and Imaging Findings

The prevalence of chest pain and breathlessness was similar between HHS and BHS: 84% vs. 82% and 31% vs. 33%, respectively. Neither clinical feature was found to be a significant risk factor for HHS. ST segment changes on the EKG were also comparable between HHS and BHS (67% vs. 70%) and were not a risk factor for HHS. The left ventricular ejection fraction values in the Stiermaier et al. study [7] were as follows: median: 43 (IQR: 31-45) for HHS and 40 (IQR: 35-45) for BHS. In contrast, Ghadri et al. [6] reported it in means with standard deviations: 40.2 ± 9.4 for HHS and 42.6 ± 11 for BHS patients. In terms of cardiac imaging findings, a significant difference was observed in the patterns of cardiac ballooning. The odds of apical ballooning occurring in HHS patients were found to be lower relative to BHS patients (OR: 0.42, 95% CI: 0.23-0.75). Conversely, the odds of midventricular ballooning occurring in HHS patients were found to be higher relative to BHS patients (OR: 2.52, 95% CI: 1.36-4.68). No significant association was found for basal ballooning between the groups. *Outcomes*

**Figure 2:**
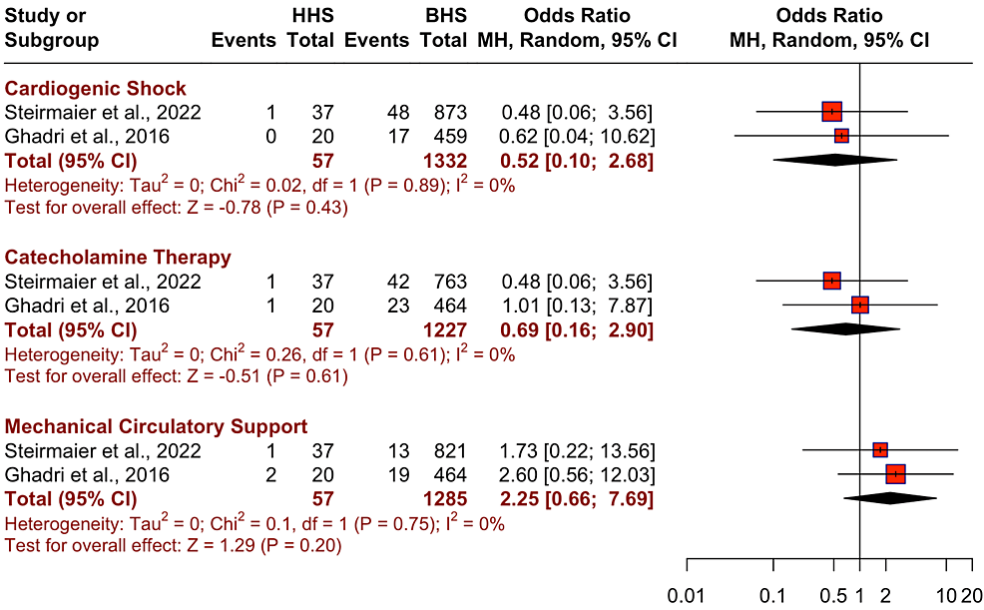
Comparison of pooled risks of outcomes in happy heart syndrome (HHS) vs. broken heart syndrome (BHS)* *[6,7]

The differences in the outcomes, including cardiogenic shock, catecholamine therapy, and mechanical circulatory support between HHS and BHS groups are illustrated in a forest plot in Figure 2. There was no significant difference in the risk for outcomes between HHS and BHS for cardiogenic shock (OR: 0.52, 95% CI: 0.1-2.68, p=0.43), catecholamine therapy (OR: 0.69, 95% CI: 0.16-2.9, p=0.61), and mechanical circulatory support (OR: 2.25, 95% CI: 0.66-7.69, p=0.2).

Discussion

Our pooled analysis and systematic review explores and compares comorbid conditions, clinical features, imaging findings, and outcomes in patients with TTC classified as two major subtypes: HHS and BHS. We found that BHS patients had a higher prevalence of comorbid diabetes mellitus and were more likely to have apical ballooning on cardiac imaging, whereas patients with HHS were more likely to have midventricular ballooning. Other comorbidities (hypertension, hypercholesterolemia, and smoking), clinical features (chest pain and breathlessness), EKG changes (ST segment changes), basal ballooning on cardiac imaging, and outcomes (cardiogenic shock, catecholamine therapy, and mechanical circulatory support) did not have a significant association between the two groups.

A growing body of literature suggests a causal link between joyful emotional triggers and Takotsubo syndrome [4,8], contradicting the prevailing hypothesis that only negative emotions precipitate TTS. Two large population-based registry analyses have been conducted to analyze the clinical features and outcomes of TTS based on the type of emotional stimulus (positive or negative), both of which have been included in our analysis [6,7].

An international collaborative systematic review of 1109 patients with TTC reported the prevalence of comorbidities as follows: hypertension in 54% (range: 27%-83%), dyslipidemia in 32% (range: 7%-59%), diabetes in 17% (range: 4%-34%), and smoking in 22% (range: 6%-49%)[9]. Our pooled findings show a similar trend, which also agrees with previous observations related to TTC [10]. However, except for diabetes mellitus, no significant association was found between comorbid conditions and HHS versus BHS subgroups of TTC. The prevalence of diabetes in TTC has been reported to range between 14% and 16% [9,11], which aligns with our findings (14.3%). By exploring the two subgroups of TTC, we found the prevalence of diabetes to be higher in those with a negative emotional trigger (14.8%) than in those with a positive trigger (3.5%). Furthermore, we found diabetes to be a significant predictor of negative emotional triggers rather than a positive one (odds of predicting HHS vs. BHS: 0.26, 95% CI: 0.07-0.94). Various mechanisms have been proposed attributing the autonomic neuropathy in diabetes, leading to a state of catecholamine depletion or hyporesponsiveness, which has been associated with a protective effect against poor outcomes in patients with TTC [12]. However, the exact pathophysiological and molecular mechanisms linking diabetes to TTC and the differential effect based on the type of emotional trigger need further exploration.

Regarding the clinical features of TTC, an analysis of the International Takotsubo Registry found the most common presenting symptoms to be chest pain (75%), dyspnea (47%), and syncope (7.7%) in all patients; however, the study did not differentiate between the subtypes of HHS or BHS [11]. Our analysis found chest pain to be most prevalent (82%), followed by breathlessness (33.5%), and a similar trend was observed in both HHS and BHS subtypes. Neither clinical feature was found to be a predictor for HHS or BHS. This highlights that although both entities have different underlying emotional triggers, the subsequent clinical presentation overlaps, and it remains unclear if this has a bearing on long-term differences in prognosis.

Electrocardiographic results revealed ST-segment elevation to be the most frequently reported (43%) finding in TTC, likely secondary to transient LV apical and/or wall dyskinesia [11,13-16]. Our analysis found a higher prevalence of ST elevation overall (70%), which was similar across both HHS and BHS. ST-segment elevation was not found to be a predictor for HHS or BHS subtypes. Cardiac imaging studies evaluating the appearance of stress cardiomyopathy have reported apical ballooning (60-80%) as a characteristic finding, followed by midventricular (14-40%) and basal hypokinesis (1-2%) [17,18]. This is in line with the prevalence noted in our study: 84% apical ballooning, 13% midventricular ballooning, and 1.8% basal ballooning. Interestingly, our pooled analysis notes apical ballooning to be a significant predictor of TTC with a negative emotional trigger (odds of HHS vs. BHS: 0.42, 95% CI: 0.23-0.75) and midventricular ballooning to be a significant predictor of TTC with positive emotional triggers (odds of HHS vs. BHS: 2.52, 95% CI: 1.36-4.68). However, basal ballooning was not found to be statistically significant.

Although many patients with stress cardiomyopathy go on to recover, existing literature based on the International Takotsubo Registry and the National Inpatient Sample supports a risk of in-hospital complications that is similar to acute coronary syndrome (19.1% versus 19.3%) [11,19]. However, our overall analysis of the two registries found lower in-hospital mortality in TTC patients (1.2%). Additionally, no in-hospital deaths were reported among patients with cardiomyopathy following a positive emotional trigger, suggesting better prognostic implications in patients with HHS compared to BHS. Outcomes of cardiogenic shock and requirement for catecholamine therapy were lower in patients with HHS over BHS (1.7% vs. 4.9% and 3.5% vs. 5.3%, respectively). However, we did note a higher use of mechanical ventilation support in patients with HHS over BHS (5.3% vs. 2.5%). Our analysis provides preliminary findings encapsulating the differential clinical features and outcomes between positive and negative emotional triggers in patients with TTC. However, larger and more long-term prospective studies are needed to further explore and establish the prognostic significance of TTC based on these two subtypes.

Limitations

This review has a few limitations. Primarily, it included only two observational studies, compounded by a small sample of HHS patients, leading to limited statistical power; underscoring the necessity of conducting larger-scale studies. Additionally, categorizing patients based on the complex and multifaceted nature of emotional triggers is inherently challenging. Most of our findings regarding comorbidities, clinical features, imaging, and outcomes did not reach statistical significance. Hence, the scarcity of data on HHS in the literature limits a comprehensive analysis. Our study does not offer insights into the pathophysiological mechanisms or the long-term prognostic implications based on positive or negative emotional triggers in TTC patients. Future prospective, long-term studies involving higher-powered, larger sample sizes must be performed to address these aspects.

## Conclusions

This pooled analysis explored the differences between HHS and BHS subtypes of TTC differentiated by positive or negative stressors. Our analysis found certain imaging disparities while highlighting similarities in comorbidities, clinical features, and outcomes. HHS has a higher predilection for midventricular ballooning patterns, while BHS more frequently shows an apical ballooning pattern. Diabetes mellitus was found to be less prevalent in HHS. Clinical features and electrocardiography lacked differentiating power, emphasizing the need for advanced imaging. This analysis provides pivotal insights into the existing body of knowledge while underscoring the need for larger-scale research to decipher the pathophysiology of these related cardiac conditions to refine diagnostic strategies for the same.

## References

[1] Singh T, Khan H, Gamble DT, Scally C, Newby DE, Dawson D (2022). Takotsubo syndrome: pathophysiology, emerging concepts, and clinical implications. Circulation.

[2] Sato H, Tateishi H, Uchida T (1990). Takotsubo type cardiomyopathy due to multivessel spasm. Clinical Aspect of Myocardial Injury: From Ischemia to Heart Failure.

[3] Ono R, Falcão LM (2016). Takotsubo cardiomyopathy systematic review: pathophysiologic process, clinical presentation and diagnostic approach to Takotsubo cardiomyopathy. Int J Cardiol.

[4] Qin D, Patel SM, Champion HC (2014). "Happiness" and stress cardiomyopathy (apical ballooning syndrome/takotsubo syndrome). Int J Cardiol.

[5] Ghadri JR, Wittstein IS, Prasad A (2018). International Expert Consensus Document on Takotsubo Syndrome (Part I): clinical characteristics, diagnostic criteria, and pathophysiology. Eur Heart J.

[6] Ghadri JR, Sarcon A, Diekmann J (2016). Happy heart syndrome: role of positive emotional stress in takotsubo syndrome. Eur Heart J.

[7] Stiermaier T, Walliser A, El-Battrawy I (2022). Happy heart syndrome: frequency, characteristics, and outcome of Takotsubo syndrome triggered by positive life events. JACC Heart Fail.

[8] Allen D, Parmar G, Ravandi A, Hussain F, Kass M (2015). Happiness can break your heart: a rare case of takotsubo cardiomyopathy after good news. Can J Cardiol.

[9] Summers MR, Lennon RJ, Prasad A (2010). Pre-morbid psychiatric and cardiovascular diseases in apical ballooning syndrome (tako-tsubo/stress-induced cardiomyopathy): potential pre-disposing factors?. J Am Coll Cardiol.

[10] Martin EA, Prasad A, Rihal CS, Lerman LO, Lerman A (2010). Endothelial function and vascular response to mental stress are impaired in patients with apical ballooning syndrome. J Am Coll Cardiol.

[11] Templin C, Ghadri JR, Diekmann J (2015). Clinical features and outcomes of Takotsubo (stress) cardiomyopathy. N Engl J Med.

[12] Ahuja KR, Nazir S, Jain V (2021). Takotsubo syndrome: does "Diabetes Paradox" exist?. Heart Lung.

[13] Chhabra L, Butt N, Ahmad SA (2021). Electrocardiographic changes in Takotsubo cardiomyopathy. J Electrocardiol.

[14] Duran-Cambra A, Sutil-Vega M, Fiol M (2015). Systematic review of the electrocardiographic changes in the takotsubo syndrome. Ann Noninvasive Electrocardiol.

[15] Gianni M, Dentali F, Grandi AM, Sumner G, Hiralal R, Lonn E (2006). Apical ballooning syndrome or takotsubo cardiomyopathy: a systematic review. Eur Heart J.

[16] Sharkey SW, Windenburg DC, Lesser JR (2010). Natural history and expansive clinical profile of stress (tako-tsubo) cardiomyopathy. J Am Coll Cardiol.

[17] Eitel I, von Knobelsdorff-Brenkenhoff F, Bernhardt P (2011). Clinical characteristics and cardiovascular magnetic resonance findings in stress (takotsubo) cardiomyopathy. JAMA.

[18] Kurowski V, Kaiser A, von Hof K (2007). Apical and midventricular transient left ventricular dysfunction syndrome (tako-tsubo cardiomyopathy): frequency, mechanisms, and prognosis. Chest.

[19] Ghadri JR, Kato K, Cammann VL (2018). Long-term prognosis of patients with Takotsubo syndrome. J Am Coll Cardiol.

